# 16-hour call duty schedules: the Quebec experience

**DOI:** 10.1186/1472-6920-14-S1-S10

**Published:** 2014-12-11

**Authors:** Charles Dussault, Nathalie Saad, Johanne Carrier

**Affiliations:** 1 2011-2012 Executive Committee of the Fédération des médecins résidents du Québec

## Abstract

Since 1 July 2012, as a result of a labour arbitration ruling in the province of Quebec and the subsequent agreement negotiated by the Fédération des médecins résidents du Québec, all 3,400 medical residents training in Quebec have been on a 16-hour duty schedule for in-house calls. This is a major change within medical teaching sites, as well as a professional and educational challenge for physicians-in-training and their supervisors. The Quebec ruling now raises similar issues for all medical residents in Canada because of its legal basis, namely the Canadian Charter of Rights and Freedoms.

## The evolution of duty hours and call duty over the last 25 years

Learning conditions and approaches have changed with the evolution of medical science over the last 25 years, and so has our knowledge of the impact of sleep deprivation.

In 1987, sleep deprivation was singled out by an American family as a major contributing factor in the death of their daughter, Libby Zion. This young woman died in March 1984 after being treated by, among others, two sleep-deprived medical residents at a time when there were no specific rules on duty hours for physicians-in-training. Following a grand jury's indictment of the two residents involved, the New York State Commissioner of Health established a blue-ribbon panel of experts headed by Bertrand M. Bell, a primary care physician. The Bell Commission evaluated the training and supervision of doctors in the state and developed a series of recommendations with regard to patient care issues, including resident duty hours. In 1989, New York State adopted the Bell Commission's recommendations that residents not be allowed to work more than 80 hours a week or more than 24 consecutive hours, and that attending physicians had to be physically present in the hospital at all times (New York State Department of Health Code, Section 405). It is important to note that, in 1991, the New York Court of Appeals unanimously ruled that the two physicians charged in the Libby Zion case – who were residents at the time of her death – should not be charged with gross negligence.

In view of this case, the Fédération des médecins résidents du Québec (FMRQ) examined its members’ work conditions and committed to changing its own collective agreement in order to reduce consecutive call duty hours in hospitals to 24, from the previous maximum of 36. In 1989, a mediation board – chaired by Judge Georges Chassé – was established in accordance with an agreement in principle signed in 1987 by the FMRQ and the Quebec Ministry of Health and Social Services to examine on-call schedules and recommend a new article pertaining to them in the FMRQ collective agreement. The Federation submitted a document stating that medical residents may not work more than 16 hours per day, including on-call schedules. After 66 hearings over a 12-month period, Judge Chassé ruled in favour of a 24-hour on-call duty system and a maximum of one call every four days. He preferred this system to New Zealand’s 16-hour call system, which he thought was still unproven at that time.

Implemented in 1990, this first step toward better working conditions for medical residents became the new norm. However, in recent years, science has provided new evidence that has compelled us to reconsider these regulations. For example, recent studies on sleep deprivation have shown a significant detrimental impact on medical residents after more than 16 continuous work hours. These studies point to a decline in cognitive function, poor clinical performance, a 36% increase in the risk of serious medical error [1], poor performance behind the wheel, twice the risk of being involved in a car accident, and a 61% increased risk of needle-stick injury after a call duty of 24 consecutive hours [2]. Furthermore, the risk of preventable medical errors is 3 to 7 times higher when a medical resident performs a call duty of 24 hours or more in a given month, as compared with 16-hour call duty schedules [3].

### Years later, a grievance contests 24-hour call duty schedules

In May 2007, 17 years after Quebec had adopted 24-hour call duty schedules, a medical resident filed a grievance against his employer – a university hospital – contesting the validity of Article 12 of FMRQ’s collective agreement concerning 24-hour call duty schedules. He argued that imposing 24 hour work schedules on an employee violated Section 7 of the Canadian Charter of Rights and Freedoms [4] and Section 1 of the Quebec Charter of Human Rights and Freedoms, as they affect the individual’s “right to life, and to personal security, inviolability and freedom [5]” and, therefore, had an impact on the integrity and security of residents and patients. He also argued that the article in question contravened Section 46 of the Quebec Charter of Human Rights and Freedoms, which stipulates that “Every person who works has a right, in accordance with the law, to fair and reasonable conditions of employment which have proper regard for his health, safety and physical well-being [5].”

The grievance was sent to arbitration. Experts were called, including Dr. Charles Czeisler, Baldino Professor of Sleep Medicine, Harvard Medical School and Senior Physician, Division of Sleep Medicine, Department of Medicine, Brigham and Women's Hospital; Dr. Elisabeth Paice, Former Dean Director at London Deanery and currently Acting Director of Medical and Dental Education with the United Kindgom’s National Health Service (NHS); and Professor Roger Godbout, a Quebec psychologist and researcher on sleep deprivation. Medical residents from different universities were also invited to testify as to their experience with 24-hour call schedules.

On 7 June 2011, over four years later, 24-hour call duty for Quebec residents was declared an unacceptable measure with regard to both charters of rights by arbitrator Jean-Pierre Lussier [6], who then made it a requirement for all Quebec training hospitals to limit work hours to 16 hours during a 24-hour period in-house. One month later, the McGill University Health Centre, where the resident had worked at the time of his grievance, challenged the ruling with regard to the 6-month deadline imposed to implement 16-hour call duty schedules in Quebec. This motion was subsequently withdrawn on 12 January 2012 (Table 1).

**Table 1:** 24-hour call duty schedules in Quebec: grievance and negotiations

9 May 2007	Submission of a grievance by a medical resident contesting the validity of the FMRQ’s collective agreement on the basis that 24-hour call duty schedules are contrary to the Canadian and Quebec charters of rights Request that call duty schedules not exceed 16 hours in a 24-hour period
30 October 2007	Grievance sent to arbitration

16 January 2009	First arbitration hearing

14 July 2009	Preliminary objections by the medical establishment

26 April 2010	Formal beginning of arbitration Medical experts, hospital authorities, and medical residents testify

24 September 2010 – 6 November 2010	Opinion survey of Quebec population on their knowledge and perception of resident work hours and conditions

7 June 2011	Arbitrator’s ruling against 24-hour call duty schedules, including implementation to be completed within 6 months (on 7 December 2011)

17 September 2011	Signing of the FMRQ’s agreement-in-principle with the Government of Quebec for a new collective agreement Implementation of 16-hour schedules to be completed by 1 July 2012

22 December 2011	Signing of the collective agreement by the Minister of Health and Social Services of Quebec, with immediate effect

1 January 2012	Launch of the FMRQ’s Grant program for research projects on reorganization of call schedules in an establishment in Quebec

1 July 2012	Deadline for implementation of 16-hour call duty schedules in Quebec

### Negotiating 16-hour call duty concurrently

While these legal proceedings were ongoing, the FMRQ entered into negotiation of its new collective agreement, including 16-hour call duty as a priority. On 22 December 2011, a new collective agreement (2010-2015) [7] was signed, making the 16-hour maximum for on-call duty a standard to be implemented as of 1 July 2012. This is aligned with practice for medical residents in the United States, the United Kingdom, Australia, and New Zealand. Perhaps most importantly, with this ruling, Quebec became the first province in Canada to initiate in-house 16-hour call duty and the first in North America to implement it in all of its teaching sites, which include over 100 medical establishments, and at all levels of residency to which in-house calls apply.

### Support from the Quebec population

In the fall of 2010, while the FMRQ was negotiating its new collective agreement, it commissioned an independent firm to survey the Quebec population on medical resident work hours [9]. Telephone interviews were conducted between 24 September and 6 November 2010 among Quebec’s general population, with a representative sample of 1,210 adults (18 years and over). The margin of error was established at 2.89%.

The results of this survey were quite telling. Eighty-four percent of respondents believed medical residents worked between 40 and 60 hours a week in hospital settings. In addition, 92% believed medical residents should never work more than 16 consecutive hours in hospitals, while only 8% advocated for a maximum of 24 consecutive hours. Seventy-two percent were of the opinion that patients should be informed if the medical resident treating them had been in the hospital for 16 hours or more.

### Changes to call duty schedules in the United States

While events were unfolding in Quebec, resident work hours remained an important issue in the United States. On 15 December 2008, the Institute of Medicine published a consensus report entitled *Resident Duty Hours: Enhancing Sleep*, *Supervision*, *and Safety*[8], designed to optimize graduate medical trainee hours and work schedules to improve patient safety. On 1 July 2011, the Accreditation Council for Graduate Medical Education (ACGME) implemented new regulations for all accredited medical training institutions in the United States that limit call duty schedules to 16 consecutive hours for first year residents. Residents in their second year and beyond still make 24-hour in-house calls, with strategic napping suggested. Training institutions that do not respect these rules risk losing their accreditation.

### Addressing the scientific evidence

Sixteen-hour call duty schedules will change how physicians in Quebec work every day and how they view their profession, as well as the way training is provided. Continuity of care, quality of training, missed procedural opportunities – an issue that is often raised by residents in surgical programs – an increased number of handovers, and the risk of moving to a shift-work mentality are among the main concerns. Quebec’s medical residents and residency programs are aware of the challenges, and it will be important to take these factors into consideration as we go forward. It will also be important to consider the effects of the increasing number of medical trainees along with the budgetary constraints that limit access to technical equipment and operating rooms. These are factors that also have a major impact on learning opportunities for medical residents, regardless of call duty schedules.

Sixteen-hour call duty schedules do not change the total number of hours worked by a medical resident (Table 2). The majority of senior residents take home calls and will continue to be available on a 24-hour basis, like the physicians training them. The 16-hour call duty schedules involve a reorganization of residents’ presence in the hospital, not a reduction in the total number of hours worked. However, it is important to remember that any work reorganization comes with challenges, and a review of teaching and monitoring approaches will need to be carried out.

**Table 2:** Comparison of number of calls, work days, hours, and gaps in call coverage for a given call schedule model with five residents in the service over a 28-day rotation period

	24-hour model	16-hour model (including call duty and evening and night shifts)
**Number of calls**	5.2 24-hour calls	5.2 16-hour calls PLUS evening and night shifts

**Attendance during the daytime**	16.2 days	15.2 days

**Total number of calls and night shift hours**	125 hours	128 hours (45 hours of evening and night shift work AND 83 hours of call duty)

**Total number of hours**	250 hours per 28-day periods OR 62.7 hours per week	244 hours per 28-day period OR 61.1 hours per week

**Total gaps in call list**	2 Friday calls (overnight)	2 Friday calls (17:00 to 23:00)

### Monitoring the change and evaluating the impact: the research continues

The FMRQ has a responsibility to protect all medical residents in Quebec and to ensure that their training and working conditions are optimal. To continue investigating the impact of the changes and to further improve working and training conditions in medicine, the FMRQ has set up a grant program to support research into various aspects of the implementation of the new 16-hour call duty [10]. Quebec medical residents are invited to submit research proposals addressing any of the following themes:

• **academic issues** such as quality of training, clinical exposure, successful completion of exams, academic models, and tools for a smooth transition to 16-hour call duty in an establishment;

• **organization of care** issues such as additional cost to the health care system and changes to departments’ operations;

• **evaluation of the implementation process**;

• **impact on medical residents’ and staff physicians’** quality of life, health, and wellness; and

• **impact on patient health and safety** with regard to quality of care delivered, continuity of care, transfer of information at handover, prescriptions, and length of stay.

The 16-hour call duty schedules are a work in progress. We must educate residents, chief residents, and program directors; develop tools and call duty models; and work with experts to develop, among other things, new teaching approaches and protocols for handovers. Doing all of this will enable us to improve and ensure continuity of care for patients, which remains a major concern among medical residents.

Medical science evolves rapidly and has demonstrated that change is necessary with regard to call duty for residents. The FMRQ will continue to be involved in the evaluation of the new work schedules and their impact on patient safety, quality of training, and any other consequences or challenges in order to make the necessary adjustments as 16-hour call duty schedules are implemented throughout Quebec.

## Authors’ contributions

All authors contributed equally to the preparation of this article.

## Competing interests

The authors declare that they have no competing interests.

## Declarations

Resources and secretariat support for this project was provided by the Royal College.

This article has been published as part of *BMC Medical Education* Volume 14 Supplement 1, 2014: Resident duty hours across borders: an international perspective. The full contents of the supplement are available online at http://www.biomedcentral.com/bmcmededuc/supplements/14/S1. This article was submitted in 2011 and peer reviewed in 2012. Final acceptance for publication as part of this supplement was in 2014.

Publication of this supplement was supported and funded by the Royal College of Physicians and Surgeons of Canada. The funding agency played no role in the design, in the collection, analysis and interpretation of data; in the writing of the manuscripts; and in the decision to submit the manuscript for publication. The articles have been through the journal's standard peer review process for supplements. The Supplement Editors declare that they have no competing interests.
